# Carbapenem Resistance in Three Hospitals in India: A Need for Strengthening Infection Prevention and Control Practices

**DOI:** 10.1017/ash.2025.200

**Published:** 2025-09-24

**Authors:** Bharat Randive, Akash Patel, Rajesh Karyakarte, Shaoli Basu, Shahzad Mirza, Sweety Singh, Mrunmayi Naik, Kavita Sangma, Sarah Fisseha, Anushruti Gupta, Sarah Sabour, Meghan Murray, Amelia S. Bhatnagar, Matthew L Robinson, Melanie Curless, Patricia J Simner

**Affiliations:** 1 B J Government Medical College-Johns Hopkins Clinical Research Site, Pune, India; 2 Johns Hopkins Center for Infectious Diseases in India, Pune, India; 3 Johns Hopkins Hospital, Baltimore, Maryland, USA; 4 Department of Microbiology, Byramjee Jeejeebhoy Government Medical College, Pune, India; 5 Department of Laboratory Medicine, P. D. Hinduja National Hospital and Medical Research Centre, Mumbai, India; 6 Department of Microbiology, Dr. D. Y. Patil Medical College, Hospital and Research Centre, Dr. D. Y. Patil Vidyapeeth University, Pune, Maharashtra, India; 7 Johns Hopkins Center for Infectious Diseases in India, Baltimore, Maryland, USA; 8 Centers for Disease Control & Prevention, Division of Healthcare Quality Promotion, Atlanta, GA, USA; 9 Department of Pathology, Johns Hopkins University School of Medicine, Baltimore, Maryland, USA

## Abstract

**Background:** Carbapenem-resistant (CR) organisms (CROs) pose a serious public health threat. We examined the burden of CRO colonization, the proportion of CROs among clinical isolates, and infection prevention and control (IPC) practices in three hospitals in India. **Methods:** This study was conducted December 2023 to December 2024 in medical intensive care units (ICUs) at three hospitals in western India. CRO colonization was assessed by direct MacConkey method in rectal/stool specimens collected ≤24 hours after admission and weekly until colonization detection or ICU discharge, colonies < 25mm from carbapenem disks were processed for bacterial identification and carbapenem susceptibility. Proportion of CROs in clinical isolates was assessed by screening Gram-negative bacilli (GNB) identified for carbapenem susceptibility. CRO was defined as GNB resistant to any carbapenem. Carbapenemase production among Enterobacterales was detected by modified carbapenem inactivation method (mCIM). Fifty day shift hand hygiene (HH) observations were collected weekly to measure adherence. Fluorescent gel markers (FGM) were placed on high-touch surfaces (HTS) to assess environmental cleaning (EC); effectiveness was assessed by proportion of FGM removed the following day. Nine key EC indicators were observed weekly to monitor cleaning technique. HH and EC data included were from June 2024 to November 2024.

The epidemiological triad model (Population-Environment-Agent) is used to describe results. **Results:** Population: Over half (476 [55%]) of 869 patients screened at ICU admission were colonized with CROs. An equal proportion of colonization was observed among patients without prior healthcare exposure in last 90 days (55% [217/396]). Of the 660 GNBs isolated from clinical specimens, 60% were CROs. **Environment:** CRO colonization was acquired by 65% (20/31) of the patients who remained in ICU for ≥ 7 days. Average HH adherence was 50% (30%-69%). HTS cleaning effectiveness averaged 65% (50%-77%). Adherence with correct EC technique was 77% (53%-86%). **Agent:** Among clinical isolates, 92% Acinetobacter-baumannii-complex, 70% Klebsiella pneumoniae, and 47% Escherichia coli were CRO. K. pneumoniae (35%) was the most frequently isolated CRO followed by A.baumannii-complex (33%) and E.coli (16%). Among colonization screening swabs, E.coli (57%) was the most frequently isolated CRO followed by K. pneumoniae (23%) and A.baumannii-complex (10%). 96% of CR Enterobacterales among clinical isolates and colonization screening were carbapenemase producers. **Conclusions:** The high prevalence of CRO colonization, acquisition rate, and carbapenem resistance indicate a high level of CRO threat in these Indian ICU settings with suboptimal IPC measures. There is an urgent need to strengthen IPC practices to interrupt transmission in healthcare settings.

